# Deciphering supramolecular arrangements, micellization patterns, and antimicrobial potential of bacterial rhamnolipids under extreme treatments of temperature and electrolyte

**DOI:** 10.3389/fmicb.2024.1493843

**Published:** 2024-11-13

**Authors:** Samia Sikandar, Asif Jamal, Afsheen Mansoor, Mounir M. Bekhit, Shakira Ghazanfar, Muhammad Ishtiaq Ali, Michael Urynowicz, Zaixing Huang

**Affiliations:** ^1^Department of Microbiology, Quaid-i-Azam University, Islamabad, Pakistan; ^2^Department of Pharmaceutics, College of Pharmacy, King Saud University, Riyadh, Saudi Arabia; ^3^National Institute of Genomics and Advanced Biotechnology, National Agricultural Research Center, Islamabad, Pakistan; ^4^Department of Civil and Architectural Engineering and Construction Management, University of Wyoming, Laramie, WY, United States; ^5^National Engineering Research Center of Coal Preparation and Purification, China University of Mining and Technology, Xuzhou, China

**Keywords:** critical micelle concentration, biosurfactant, rhamnolipid, thermodynamic parameters, micellization, electrolytes

## Abstract

The micellization properties of rhamnolipids (RLs) in extreme electrolyte concentrations and temperatures have gained considerable attention due to their broad industrial applications. In this study, the aggregation behavior, specifically the micellization pattern (critical micelle concentration (CMC)) of RLs produced from a newly isolated thermophilic strain of *Pseudomonas aeruginosa* from a harsh environment of an oil field, was investigated by a spectrophotometric method at various temperatures (293–393 K) and electrolyte concentrations (NaCl: 2–20%). The result indicated that the *CMC* values (0.267–0.140 mM⋅dm^−3^) were both electrolyte- and temperature-dependent exhibiting a U-shaped trend as temperature and NaCl concentration increased. Variations in NaCl concentration and temperature also affected the standard Gibbs free energy (Δ*G^o^_mic_*), enthalpy (Δ*H^o^_mic_*), and entropy (Δ*S^o^_mic_*) of micellization. The molecule also showed stability at a broad range of temperatures, pH, and NaCl concentrations. Thin-layer chromatography (TLC) and Fourier-transform infrared (FTIR) analysis confirmed the similarity in composition between the crude extract and the commercial RL with Rf values of 0.72 for mono-rhamnolipids and 0.28 for di-rhamnolipids. FTIR analysis confirmed the chemical nature particularly key aliphatic functional groups present in the fatty acid tail of RLs and the -COC- bond in the structure of the rhamnose moiety. Additionally, LC-ESI-QTOF analysis confirmed corresponding ionic fragments of mono- and di-rhamnolipids congeners. Furthermore, the antimicrobial potential was determined against different human pathogens in the absence and presence of NaCl by measuring zones of inhibition. The result revealed enhanced inhibitory effects against Gram-positive pathogens (*S. aureus*, *S. epidermidis*, and *L. monocytogene*), with zones of inhibition of 26, 30, and 20 mm in the presence of NaCl. These findings underline the role of NaCl in the micellization of RL molecules and highlight their importance in environmental applications, pharmaceuticals, and various life science sectors.

## Graphical abstract

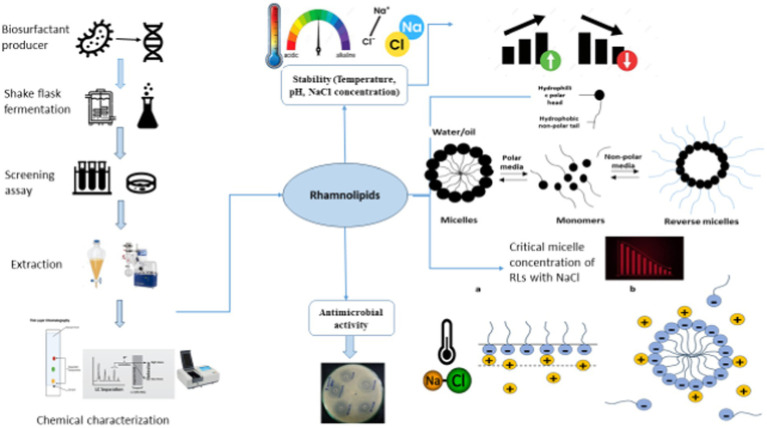

## 1 Introduction

The surface interaction aspect of science is primarily centered on the micellization behavior and thermodynamic characteristics of surface-active metabolites whether they are of synthetic or natural origin (Li, 2017; Paulino et al., 2016). The broad commercial potential and industrial applications have stimulated the dendritic growth of bacterial glycol-conjugates (Liley et al., 2017; Oulkhir et al., 2023). Of particular interest, the synthesis of thermodynamically stable surfactants has spurred new advancements in the field of nanotechnology. Consequently, based on the fundamental principles of self-assembly, the number of technical applications and surfactant products has been growing rapidly (Shalini et al., 2017; Xu et al., 2018; Rehman et al., 2021).

Rhamnolipids (RLs), a class of naturally occurring surface-active molecules, composed of hydrophilic two rhamnose molecules joined with hydrophobic one or two *β*-hydroxydecanoic acids, have gained ecological significance owing to their multifaceted characteristics, diverse biotechnological applications, and versatile physicochemical properties (Zhao et al., 2015; Abdel-Mawgoud et al., 2010). The biosynthesis of surfactants is associated with various functions including changing surface properties, enhancing the bioavailability of non-polar substrates, contributing to biofilm development, regulating microbial signaling, and influencing physio-chemical interactions (Rikalovic et al., 2015; Henkel et al., 2012). Rhamnolipids in an aqueous medium exhibit versatile surface behavior by adsorbing at the interface and monomers tend to self-assemble as a result of repulsive forces in the aqueous system producing micelles, bilayers, and vesicles beyond a specific surfactant concentration referred to as critical micelle concentration (CMC) (de Araujo et al., 2016; Hassan et al., 2016). The formation of micelle allows a reduction in surface and interfacial tension (Bharali et al., 2013). These micelles are thermodynamically stable and exhibit geometrically organized structures imparting unique properties to the surfactants (Dobler et al., 2016).

Understanding surfactant behavior, particularly at the CMC, is crucial for advancements in surface science and technology. Hydrophobic interactions are key to micelle formation. Research has been focused on understanding the phase behavior of surfactants in different media, and remains the study interest in this field (Kłosowska-Chomiczewska et al., 2017; Mańko et al., 2014). In an aqueous system, surfactants align at the air–water interface to reduce the free energy and allow micelle formation. The extensive association of micelle aggregate depends on the chain length of the hydrophobic part of the biosurfactant (Paria et al., 2015; Kitamoto et al., 2009). The adsorption of surface-active biomolecules at the interface results in a reduction in surface tension. RL aggregates at low concentrations attain a variety of shapes such as flexible rigid ellipsoidal, spheres, spirals, rods, lamellar sheets, and bilayer vesicles depending on the molecular composition, and can form larger crystalline structures at high concentrations (İkizler et al., 2017).

RLs produced by *Pseudomonas* species offer several advantages including low toxicity, high degradability, and stability across a wide range of environmental conditions including temperature, pH, and salinity (Rehman et al., 2021; Cerqueira dos Santos et al., 2024). The current study focuses on RLs produced by *Pseudomonas* isolated from an oil field, where the bacteria thrived on hydrocarbons as the sole carbon source. *Pseudomonas* species show similarities with extremophiles because of their metabolically versatile nature and the ability to adapt to various environments (Craig et al., 2021; Raiger Iustman et al., 2015). They are halotolerant and thermotolerant known for their versatility in utilizing alkanes and other water-soluble compounds by producing RLs (Kumar et al., 2008; Mishra et al., 2017). RLs produced by *P. aeruginosa* are particularly stable and effective for the removal of aromatic and hydrophobic contaminants found in soil (Raiger Iustman et al., 2015; Yalçın and Ergene, 2009). These RLs exhibit unique biochemical properties characterized by multiple functional groups and chirality centers. They show heterogeneous structures and possess several advantages including biodegradability with lower toxicity, a lower critical micelle concentration, higher surface activity, slow adherence, and the ability to form molecular assemblies and liquid crystals (Shi and Xia, 2003; Lovaglio et al., 2015). Moreover, the recent understanding of rhamnolipids and their interfacial chemistry has reshaped our conventional understanding of amphiphilic water systems. The linear hydrocarbon chains present in glycolipids provide excellent flexibility for manipulations to achieve desired physical properties (Yang et al., 2020). A comprehensive understanding of the physiochemical dynamics of biosurfactant solutions is crucial for both theoretical and applied research (Zhang et al., 2022).

Self-assembled rhamnolipids are promising in industrial and biotechnological processes even in harsh conditions. Micelles that can maintain their stability at elevated temperatures are useful in processes like bioremediation, enhanced oil recovery, and enzymatic reactions. Their ability to solubilize hydrocarbons under extreme pH levels, high salinity, and high temperature enhances their uses in the extraction of heavy metals, oil, and other hydrocarbons (Roy, 2018; Markande et al., 2021). Additionally, micelles can encapsulate sensitive molecules such as drugs and enzymes, protecting them from degradation in harsh conditions like high salinity or acidic/basic environments (Drakontis and Amin, 2020). The emulsification and dispersion properties can reduce surface tension in extreme conditions, making them useful in food, petrochemical, and pharmaceutical industries. Engineered RL micelles with enhanced stability and tunable sizes could be applied to industries such as geothermal energy production and deep-sea oil recovery, where maintaining their structural integrity and functionality is important (Sarubbo et al., 2022). Additionally, hybrid micelles that are formed by combining biosurfactants with nanoparticles can improve catalytic performance under high temperatures, making them more cost-effective and environmentally friendly across various industrial applications (Liu et al., 2020; Lang and Wagner, 2017). RLs have been demonstrated antimicrobial activity against various human and plant pathogens, including *B. cereus*, *L. monocytogenes, S. aureus, E. coli, P. aeruginosa, Salmonella typhi*, *L. monocytogenes,* and *Candida albicans* (Pirog et al., 2019; Zhao et al., 2022; Magalhães and Nitschke, 2013). However, challenges remain in using RLs owing to a limited understanding of their stability under different electrolytes and solvents. Research into biosurfactants and electrolytes interactions has explored factors including hydrogen bonding, hydrophobic interactions, and hydrodynamic and thermodynamic properties, but further understanding is needed (Mishra et al., 2023).

This study seeks to address these gaps by investigating the combined effect of temperature and electrolyte (NaCl) concentrations on the CMC and thermodynamic properties of RLs. While previous investigations have predominantly explored RL behaviors on single-parameter analyses, industrial formulations often encounter various co-chemicals and electrolytes, making it crucial to understand the combined effects of temperature and electrolytes on the micellization and surface behavior of biosurfactants. Such understanding is of significant commercial and practical relevance in the field of medicine as antimicrobial agents. This research explores the phase behavior and micellization patterns of RLs produced from *P. aeruginosa* isolated from crude oil-contaminated soil under simultaneous NaCl and temperature treatments. The antimicrobial potential of RLs in the presence of NaCl was also evaluated against human bacterial pathogens. To the best of our knowledge, no previous research has comprehensively addressed the phase behavior of RLs under the combined effect of electrolytes and temperature, making this study a valuable contribution to the broader understanding of microbial surfactants and their applications in various industries. By studying RL behavior in these contexts, this research aims to expand the potential applications of RLs in environments that require high stability and adaptability, particularly in sectors that involve extreme pH, salinity, or temperature conditions. The findings could lead to improved processes in bioremediation, oil recovery, and drug formulation, showcasing the versatility and industrial relevance of RLs.

## 2 Materials and methods

### 2.1 Phylogenetic identification of bacterial isolate

The pure thermophilic bacterial isolate R3 M05PSSI, extracted from crude oil-contaminated soil in the Fimkassar oil field Chakwal, Pakistan was used in this study. The selected bacterial strain with a better production profile up to 45°C was previously screened for biosurfactant production qualitatively and quantitatively, but the appropriate research was not done yet. The selected strain was subjected to identification on a molecular basis by 16 s rRNA sequencing. The DNA was first extracted from the pure culture and amplified by PCR. The sequences obtained were then subjected to BLAST, which is a tool that identifies the homologs based on sequence similarity. The isolated strain showed maximum sequence similarity with accession ID MF612185.

### 2.2 Screening assays for confirmation of biosurfactant production

The isolated strain was previously screened for biosurfactant production, but the appropriate research work was not done. So, to confirm the production of biosurfactants in culture media different screening assays were accomplished on a cell-free broth to confirm the biosurfactant presence. Biosurfactant production was confirmed by growing the bacterial strain aerobically in a mineral salt medium (MSM) with the following composition in 1 L: K_2_HPO_4_ 10 g, NaH_2_PO_4_ 5 g, (NH_4_)_2_SO_4_ 2 g, MgSO_4_•7H_2_O 0.2 g, CaCl_2_•H_2_O 0.001 g, FeSO_4_•7H_2_O 0.008 g, and glucose 20 g. After 4 days of incubation at 37°C with an agitation rate of 50 rpm, the culture broth was centrifuged to obtain a cell-free supernatant which was further used for the detection of surface-active compounds. These assays include surface tension measurement (SFT), oil displacement assay (ODA), emulsification index (EI_24_%), drop collapse test, hemolytic assay, CTAB blue agar assay, and phenol sulfuric acid test (Wei et al., 2005; Płaza et al., 2006; Aziz et al., 2014).

### 2.3 Production and physical characterization of biosurfactants

The optimized MSM with the following composition in 1 L: Glycerol 20 mL, Biodiesel waste glycerol from *Ricinus communis* 10 mL, Biodiesel waste glycerol from *Pongamia pionnata* 10 mL, bacteriological peptone 1 g, K_2_HPO_4_ 10 g, NaH_2_PO_4_ 2.5 g, NaNO_3_ 3 g, FeSO_4_•7H_2_O 0.01 g, MgSO_4_•7H_2_O 0.05 g, CaCl_2_•2H_2_O 0.1 g, and NaCl 0.3 g was used for the biosurfactant production. After incubation for 96 h at 37°C and 150 rpm, the supernatant was obtained by centrifugation of the culture media at 10,000 rpm at 4°C for 20 min. The filtered supernatant was used to determine the emulsification index and surface tension with different physical parameters, including temperature (5–121°C), pH (Li, 2017; Paulino et al., 2016; Liley et al., 2017; Oulkhir et al., 2023; Shalini et al., 2017; Xu et al., 2018; Rehman et al., 2021; Zhao et al., 2015; Abdel-Mawgoud et al., 2010; Rikalovic et al., 2015; Henkel et al., 2012; de Araujo et al., 2016; Hassan et al., 2016; Bharali et al., 2013), and salt concentrations (NaCl 2–20%) to evaluate the stability of biosurfactant produced by the strain R3 M05PSS1. The CFS samples were incubated at varying temperatures then cooled at room temperature and checked for EI_24_% and surface tension measurement. The cell-free supernatant was also checked for EI_24_% and surface tension after autoclaving at (121°C for 20 min). The effect of pH was also checked for acidic and basic pH ranges. For this purpose, the pH of the cell-free supernatant was adjusted by using 1 N HCl and 1 N NaOH. The solutions having different pH were then incubated at 37°C for 1 h. After that, EI_24_% and surface tension were measured. To evaluate the effect of NaCl on the stability of rhamnolipids, the culture-free broth was treated with different NaCl concentrations. After treatment, the EI_24_% and surface tension were measured for each sample. The experiments were run in triplicates.

### 2.4 Extraction and chemical characterization of rhamnolipids

The crude metabolite was extracted using a solvent extraction method proposed by Bordas et al. (2005) with the acid precipitation method for compound extraction. The filtered cell-free broth was acidified to pH 2–3 with 5 N HCl to precipitate the biosurfactant overnight at 4°C. The precipitate was collected by centrifugation and solvent extraction. The extracted compound was dried in a fume hood and characterized. The crude rhamnolipid was analyzed through TLC with different ratios of the solvent system for the separation of RL-1 and RL-2. The mobile phase contained chloroform (85%), methanol (10%) and acetic acid (5%). The TLC plate was visualized under a UV lamp at 254 nm for florescence-quenching spots and 365 nm for florescent spots. The chemical structure of RLs was analyzed by FTIR spectroscopy to elucidate the chemical nature of bonds and functional groups present in the extracted rhamnolipid. Spectra for each compound was recorded in the range 400–4,500 cm^−1^ (Ali et al., 2016, 2017). The spectrum of the crude product was compared with the (control) standard rhamnolipid acquired from Sigma-Aldrich.

Biosurfactants produced from *P. aeruginosa* R3 M05PSS1 and their respective (control) standard rhamnolipids were analyzed through ultra-high-performance liquid chromatography coupled with electrospray ionization quadrupole time-of-flight mass spectrometry (UHPLC/ESI-QTOF-MS, Dionex Ultimate 3,000 and Bruker Compact). Samples were analyzed in both positive and negative ionization modes in 100–2000 m/z range. Chromatographic separation was performed using a C18 column (Waters Acquity UPLC HSS T3, 100 Å, 1.8 μm, 2.1 × 100 mm) with a gradient of mobile phase A (0.1% acetic acid in ultrapure water) and mobile phase B (0.1% acetic acid in acetonitrile) at a flow rate of 0.3 mL/min. The data was processed using the Bruker Data Analysis software, Bruker Daltonik version 5.1, and Compound Crawler version 3.1. Additionally, direct infusion MS in positive ionization mode was performed on both commercial rhamnolipids (control) and crude rhamnolipids from *Pseudomonas aeruginosa* MF612185.

### 2.5 Critical micelle concentration determination at different conditions

CMC of rhamnolipids was determined using a UV–visible spectrophotometer-1700 Pharma spectrometer (Shimadzu, Japan) under various temperature and electrolyte conditions individually and combined. CMC is the concentration of rhamnolipid at which micelles start to form. For this purpose, a known 6.25 mM stock solution of rhamnolipid was prepared in 10 mL MilliQ water. The other solutions were then prepared by diluting the rhamnolipid stock. Absorbance was measured in the UV range of 200–800 nm for different biosurfactant concentrations. A graph was plotted between absorbance and biosurfactant concentrations to determine the CMC of rhamnolipids using OriginPro 8.5 software. The effect of temperature on CMC was investigated with the temperature range of (293–393 K). The rhamnolipid stock solution was prepared and treated with a range of temperature (293–393 K) for 1 h. After that, the desired dilutions were obtained by adding the deionized water to the stock solution. The dilutions were treated at a desired temperature range in a water bath for 1 h. The surfactant solution was set for 24 h before use. Absorbance was measured for each dilution in triplicate and a graph was plotted to calculate the CMC. Similarly, the same protocol was applied to investigate the effect of electrolyte (NaCl) on the CMC. The effect of NaCl on CMC was determined by adding NaCl (2–20%) to the biosurfactant solution. The combined effect of temperature and salt was also investigated using the absorption data to calculate CMC values.

### 2.6 Determination of antibacterial activity

Antimicrobial assays were conducted with Gram-positive and negative bacteria obtained from ATCC, including Gram-negative: *Klebsiella pneumoniae* (ATCC-7881), *Salmonella typhimurium* (ATCC-14028), *E. coli* (ATCC-10536), and *Pseudomonas aeruginosa* (ATCC-10145), and Gram-positive *Staphylococcus aureus* (ATTC-6538), *Staphylococcus epidermidis* (ATCC12228), *Bacillus spizizenii* (ATCC-6633), and *Listeria monocytogenes* (ATCC-7644). The antimicrobial activities were evaluated by applying a well-diffusion method using different concentrations of RLs (Magalhães and Nitschke, 2013; Govindammal and Parthasarathi, 2013). The turbidity of the test strains was compared with 0.5 McFarland standard and cultures were produced on Muller–Hinton agar plates. Wells of 6 mm were punched with a sterile cork borer. Biosurfactant solutions (20, 40, 60, 80, and 100 μg/mL) prepared in methanol solvent were assayed in each well. After 24–48 h of incubation at 37°C, the diameter of inhibition zones was measured. The combined effect of NaCl and RLs was also recorded by well diffusion assay MHA plates, and for this purpose, NaCl was used in combination with rhamnolipids. The concentration of the stock solution for activity was selected as 100 μg/mL of rhamnolipid and NaCl (250–1000 mM). The results were observed after 24 h of incubation.

## 3 Results

### 3.1 Identification of the microbial strain

**Figure 1:**
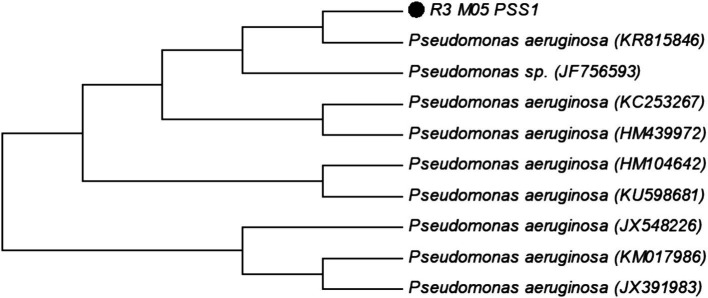
Molecular phylogenetic analysis of strain R3 M05PSS1 of *Pseudomonas aeruginosa* accession number (MF612185) by the maximum likelihood method.

The bacterial strain was molecularly identified through the characterization of conserved 16 s rRNA sequences. Similarity analysis was conducted using the BLAST search tool to identify the closest phylogenetic homolog of the isolated strain. The strain R3 M05PSS1 was classified as *Pseudomonas aeruginosa* (Figure 1).

### 3.2 Characterization of biosurfactants

The supernatant of the culture after 96 h of incubation was used to investigate the presence of surface-active metabolites with surface tension measurement being a key confirmatory assay. The surface tension of the culture by *P. aeruginosa* decreased to 29 mNm^−1^ corroborated by a positive drop collapse test. The emulsification index (EI_24_%) was also determined using kerosene oil as a standard yielding an 82.5% emulsion index. Additionally, an oil displacement assay was performed to evaluate the metabolic capability of the strain in producing bio-emulsifiers. The result showed that *P. aeruginosa* displaced the thin crude oil layer from the surface of the water for up to 7.1 cm. The phenol sulfuric acid test usually performed to determine the sugar moiety or glycolipid in the sample showed a positive result by changing color from yellow to orange confirming the presence of carbohydrates in the surfactant. Furthermore, in the CTAB methylene blue agar assay, a dark blue zone was observed confirming the anionic nature of the biosurfactant. In the blood hemolysis test, a positive result was shown by producing a clear zone of blood hemolysis around the site of inoculation after 48 h of incubation.

### 3.3 Stability of biosurfactant under temperature, pH, and salt concentration

**Figure 2:**
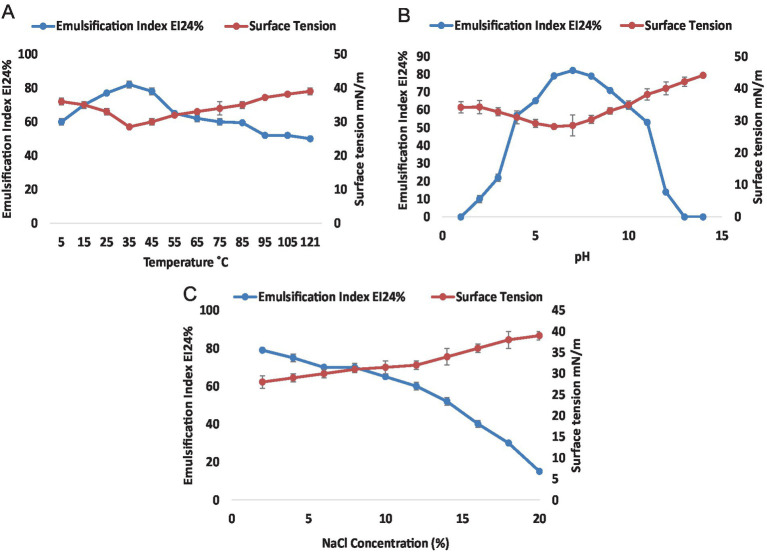
Stability of Rhamnolipids from *P. aeruginosa* strain R3M05 PSS1. **(A)** Effect of temperature (5–121°C) on the stability of rhamnolipid from *P. aeruginosa* strain R3M05 PSS1 by EI_24_% and surface tension measurement. **(B)** Effect of pH (1—14) on the stability of rhamnolipids from *P. aeruginosa* strain R3M05 PSS1 by EI_24_% and surface tension measurement. **(C)** Effect of NaCl (2–20%) concentration on the stability of rhamnolipids from *P. aeruginosa* strain R3M05 PSS1 by EI_24_% and surface tension measurement.

The physical stability of the surface-active metabolites was analyzed under different conditions including temperature, pH, and salt concentrations (Figure 2). Emulsification index and surface tensions were calculated for each varying parameter to assess the physical stability and surface-active behaviors of the biosurfactant. The effect of temperature on the stability of crude RLs indicated that these molecules were stable over a wide temperature range. It was clearly shown that temperature had no significant effect on the surface-active and emulsification properties even at high temperatures (Figure 2A). The maximum emulsification was observed at 35°C reaching 82% with a subsequent reduction to 65% at 55°C. Thereafter, EI_24_% remained almost constant up to 121°C. The surface tension remained stable across a wide temperature range from 5°C to 100°C reaching its nadir at 28.5 mNm^−1^ at 35°C. Remarkably, these metabolites remained stable even at an extreme temperature of 121°C with a 52% emulsification index and a surface tension of 39 mNm^−1^. The impact of pH on the stability of RLs was also evaluated over a pH range of 1–14 (Figure 2B). Colloidal behavior and surface-active properties were analyzed through the estimation of emulsification index and surface tension measurement. The RL demonstrated stability over a wide range of pH from 4 to 11. Notably, the emulsion index remained above 50% for pH 4–11 with the highest emulsification recorded at pH 7 (EI_24_% = 82%). Surface tension was effectively reduced from 72 mNm^−1^ to 28 mNm^−1^ at pH 6. When the supernatant was treated at pH 4–11, only minor fluctuations were observed at extreme pH. However, no emulsification was observed at both acidic and basic extremes indicating that pH has diverse effects on the surface properties of the biosurfactant, potentially altering the chemical structure and interactions of rhamnolipid molecules. Furthermore, the stability of rhamnolipids was assessed in the presence of NaCl (Figure 2C). The results indicated a decrease in RL stability with an increase in salt concentration. The highest emulsification of 79% was observed at 2% NaCl while the lowest of 15% emulsification was recorded at 20%. The lowest surface tension of 28 mN·m^−1^ at 2% NaCl was observed, suggesting that lower NaCl concentrations were more effective in maintaining stability.

### 3.4 Chemical structure and composition of biosurfactants

**Figure 3:**
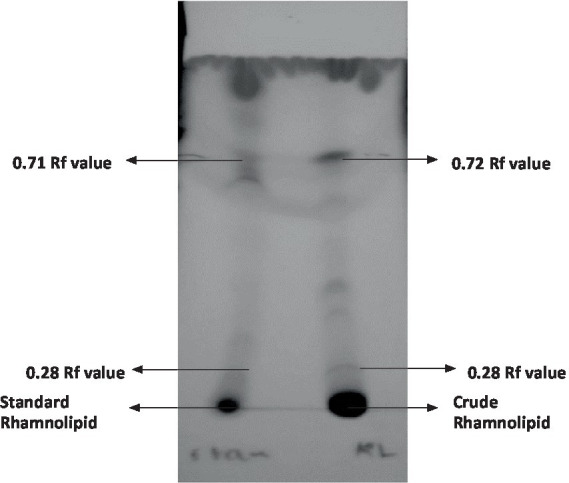
Thin layer chromatography of rhamnolipids from *Pseudomonas aeruginosa.*

The qualitative detection of the crude extract was carried out using the thin layer chromatography (TLC) technique as shown in Figure 3. The results suggest that the crude extract had a similar composition to standard rhamnolipids from Sigma-Aldrich. Positive results were observed in the crude extract from *P. aeruginosa* showing two spots of mono- and di-rhamnolipids which confirmed the similarity in composition with the standard rhamnolipids. The rhamnolipids spots with Rf values of 0.72 for mono-rhamnolipids and 0.28 for di-rhamnolipids were observed under UV.

**Figure 4:**
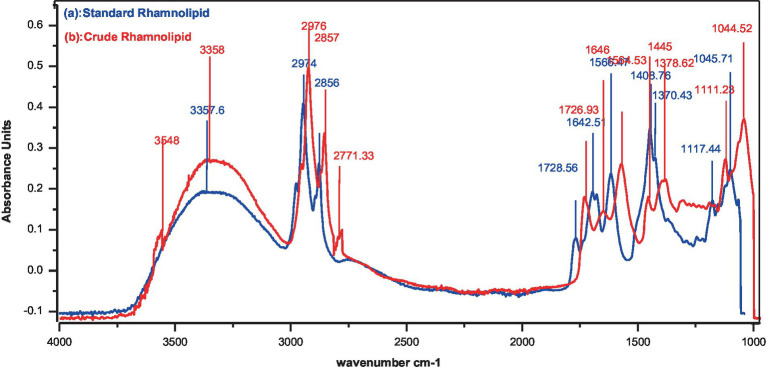
FTIR analysis of spectrum. (a) Standard rhamnolipids (control), (b) Rhamnolipids extracted from *P. aeruginosa* strain R3 M05PSS1.

FTIR analysis was conducted to confirm the chemical nature and specific functional groups in the molecule (Figure 4). It was confirmed that rhamnolipids were present in the partially purified crude compounds. Major peaks for various functional groups were detected while absorbance bands and frequencies of different functional groups have shown a correlation (Figures 4A,B). Both crude and standard rhamnolipids displayed similar bands at 3357, 2974, 2,924, and 2,856 cm^−1^ due to symmetric C-H stretching and vibration of aliphatic groups predominantly present in the fatty acid tail of hydroxydecanoic acid of rhamnolipids. The region from 2,700 cm^−1^ to 3,300 cm^−1^ showed hydrogen bonding of OH groups and stretching of C-H for CH_2_ and CH_3_ groups. The band at 1642 cm^−1^ was attributed to CH vibration while the band between 1,239 cm^−1^ and 1,457 cm^−1^ represented CH and O-H deformation vibrations. The absorption band around 1,045 cm^−1^ confirmed the structure of C-O-C groups with -COC- vibration confirming the presence of bonds between carbon and hydroxyl groups in the structure of the rhamnose moiety. The region below 1,200 cm^−1^ represented different C-H, C-O, and CH_3_ vibrations. The band at 1656 cm^−1^ indicated the C=O sugar moiety while the absorption frequency at 1728 cm^−1^ was due to the presence of carboxylic groups and ester bonds. The FTIR analysis concluded that the crude compounds contained sugar molecules as evidenced by the comparison of the spectra of the crude extract compounds with standard rhamnolipids.

**Figure 5:**
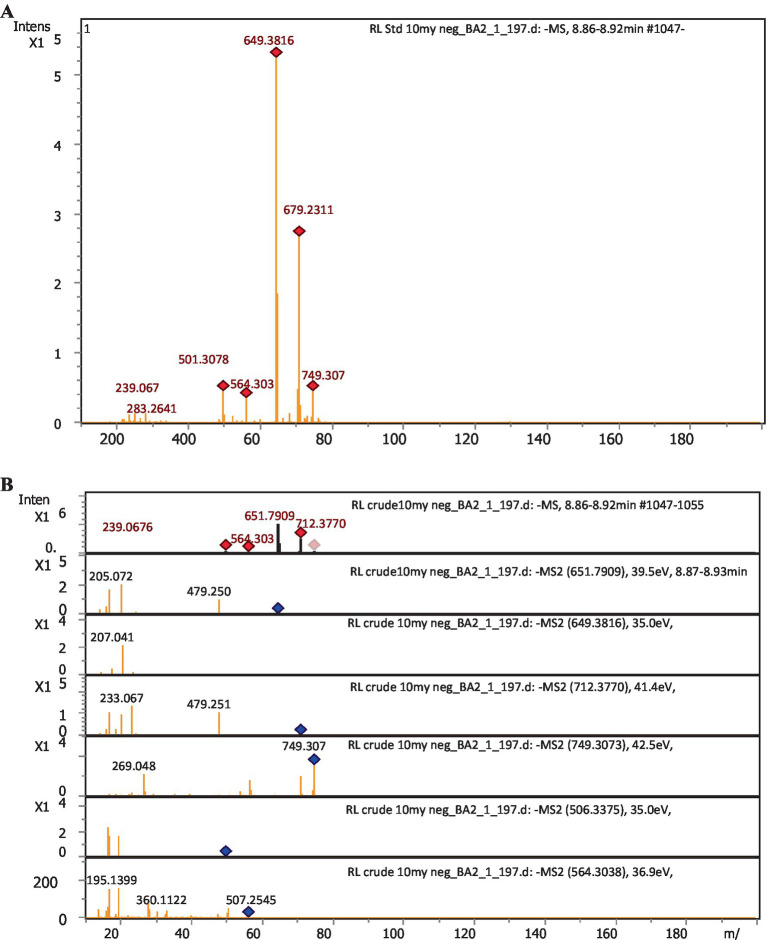
LC-ESI-QTOF-MS profile of rhamnolipids. **(A)** Sigma-Aldrich standard (control); **(B)** Extract from *P. aeruginosa* strain R3 M05PSS1.

The chemical composition of rhamnolipids synthesized by *P. aeruginosa* was compared to pure standard Sigma (99.5%) Aldrich standard through LC-ESI-QTOF mass spectrometric analysis. The standard rhamnolipid molecule exhibited major peaks at 360.1127, 506.2021, 564.30.38, 651.7932, 679.2311, and 749.3073 m/z. These peaks correspond to pseudo-molecular ions of Rha-Rha-C_10_-C_12_, Rha-Rha-C_12:1_, Rha-Rha-C_10_-C_12_-CH_3_, Rha-Rha-C_10_-C_10_, Rha-Rha-C_12_-C_10_, and Rha-Rha-C_10_-C_16_. In addition to these major peaks, minor quantities of mono- and di-rhamnolipid congeners were also detected due to the impurity (Figure 5A). In the case of the extracted rhamnolipid metabolite from *P. aeruginosa,* the mass spectrum showed corresponding ionic fragments of mono- and di-rhamnolipid congeners in the chromatogram. Among the various congeners, the major peaks corresponded to predominant protonated molecular ions, such as Rha-Rha-C_12:1_ (506.3375), Rha-Rha-C_12_ (507), Rha-C_12:2_ (360.1122), Rha-Rha-C_10_-C_12_ (564.30), Rha-Rha-C_10_-C_10_ (649.3816), Rha-Rha-C_10_-C_12_-CH_3_ (651.7909) Rha-Rha-C_12_-C_14_ (712), and Rha-Rha-C_10_ (479.2512) were detected, as shown in Figure 5B (see Table 1).

**Table 1:** Chemical structure identification and relative abundance (%) of rhamnolipid congeners using LC-ESI-QTOF-MS.

Rhamnolipid congeners	Pseudo-molecular ion fragment (*m/z*)	Relative abundance (%)	Ionic fragments
Rha-C_12:2_	360.1122	3	239.0676, 205.0720, 207.0416, 233.0678, 269.0482, 195.1399
Rha-Rha-C_12:1_	506.3375	10	
Rha-Rha-C_12_	749.3073	10	
Rha-Rha-C_10_-C_12_	564.30	10	
Rha-Rha-C_10_-C_10_	649.3816	22	
Rha-Rha-C_10_-C_12_-CH_3_	651.7909	26	
Rha-Rha-C_10_	479.2512	5	
Rha-Rha-C_12_-C_14_	712	14	

### 3.5 CMC of rhamnolipids

**Figure 6:**
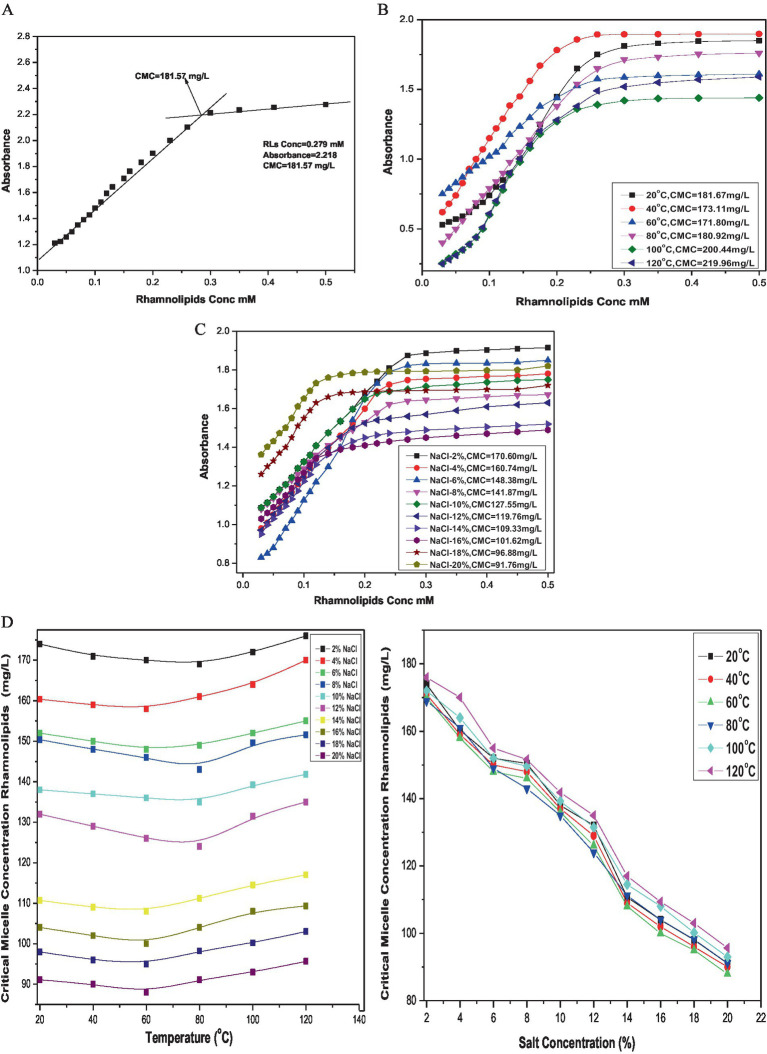
**(A)** CMC of rhamnolipids extracted from *P. aeruginosa* strain R3 M05PSS1. **(B)** CMC of rhamnolipids extracted from *P. aeruginosa* strain R3 M05PSS1 at temperature range 20°C —120°C. **(C)** CMC of rhamnolipids extracted from *P. aeruginosa* strain R3 M05PSS1 with NaCl concentration 2%--20%. **(D)** CMC of rhamnolipids extracted from *P. aeruginosa* strain R3 M05PSS1 with NaCl concentration 2–20% and varying temperature.

The CMC of the rhamnolipid was calculated from the inflection point in the absorbance versus concentration plot of the biosurfactant. The breakpoint in the absorbance at the UV wavelength of 263 nm corresponding to the transition from monomers to micelles was observed at a concentration of 181.57 mg/L at room temperature (Figure 6A). Representative plots of absorbance against rhamnolipid concentration in aqueous solution at temperature range from 20°C to 120°C and salt concentration from 2 to 20% are shown in (Figures 6B,C). The results showed the stability of the surface behavior of rhamnolipids under extreme temperatures and salt conditions. These conditions had diverse effects on the structure of rhamnolipids leading to variations in structure–function relationships. Initially, a decrease in CMC values of rhamnolipids was observed with an increase in temperature, but with further temperature increase the CMC value was also increased. The simplest class of self-association pattern is micelle formation which is significantly affected by the presence and absence of additives. For rhamnolipids, it was observed that the values of CMC decreased with the increase in NaCl concentration reaching a minimum of 91.76 mg/L at 20% NaCl. An effective strategy for reducing the CMC of surfactants is the addition of salt into the surfactant solution. Anionic rhamnolipids typically showed strong repulsive forces between their head groups and resisted efficient combinations. The plots of absorbance against the concentration of rhamnolipids in the presence of different NaCl concentrations at a temperature range of 20–120°C are presented in Figure 6D. The CMC values for rhamnolipids in the presence of electrolytes at various temperatures calculated from spectrophotometric measurement are shown in Table 2. The CMC of biosurfactant solution with added electrolyte was determined from the breakpoint in the absorbance plots. It was noted that the breakpoint became less well-defined with an increase in electrolyte concentration. Micelle formation of the surfactant could not be detected beyond a certain level of electrolyte, i.e., 20%. A decrease in the CMC of rhamnolipids was recorded with an increase in NaCl concentration and temperature.

**Table 2:** Thermodynamic parameters: CMC values, mole fraction, and thermodynamic parameters.

NaCl	Temperature (K)	CMC (mM)	X_CMC_	Δ*G^o^_mic_* (kJ·mol^−1^)	Δ*H^o^_mic_* (kJ·mol^−1^)	Δ*S^o^_mic_* (kJ·mol^−1^ K^−1^)
2%	293	0.267	0.004754	−13.0294	−0.056	0.0442
	313	0.264	0.004701	−13.9481	−0.064	0.0443
	333	0.262	0.004666	−14.8602	−0.073	0.0444
	353	0.26	0.004632	−15.7751	−0.082	0.0444
	373	0.265	0.004719	−16.6101	−0.092	0.0442
	393	0.270	0.004808	−17.4399	−0.102	0.0441
4%	293	0.246	0.004359	−13.2407	−0.434	0.0437
	313	0.244	0.004324	−14.1656	−0.495	0.0436
	333	0.243	0.004307	−15.0821	−0.561	0.0436
	353	0.247	0.004377	−15.9402	−0.630	0.0433
	373	0.252	0.004465	−16.7815	−0.704	0.0431
	393	0.262	0.004642	−17.5547	−0.781	0.0428
6%	293	0.234	0.004123	−13.3767	−0.116	0.0452
	313	0.232	0.004088	−14.312	−0.133	0.0453
	333	0.228	0.004017	−15.2745	−0.150	0.0454
	353	0.229	0.004035	−16.1791	−0.169	0.0453
	373	0.234	0.004123	−17.0292	−0.189	0.0451
	393	0.238	0.004193	−17.8873	−0.209	0.0449
8%	293	0.231	0.004042	−13.4248	−0.056	0.0456
	313	0.228	0.003993	−14.3727	−0.064	0.0457
	333	0.224	0.003924	−15.3399	−0.073	0.0458
	353	0.220	0.003854	−16.3139	−0.082	0.0459
	373	0.230	0.004028	−17.1009	−0.091	0.0456
	393	0.233	0.004081	−17.9757	−0.101	0.0454
10%	293	0.213	0.003706	−13.6362	−0.151	0.0460
	313	0.211	0.003671	−14.5914	−0.172	0.0460
	333	0.210	0.003654	−15.5369	−0.195	0.0460
	353	0.208	0.003619	−16.4980	−0.219	0.0461
	373	0.214	0.003723	−17.3449	−0.244	0.0458
	393	0.218	0.003793	−18.2146	−0.271	0.0456
12%	293	0.202	0.003508	−13.7697	−0.139	0.0465
	313	0.199	0.003443	−14.7586	−0.158	0.0466
	333	0.194	0.003357	−15.7719	−0.179	0.0468
	353	0.192	0.003322	−16.7495	−0.202	0.0468
	373	0.202	0.003495	−17.5415	−0.225	0.0464
	393	0.207	0.003581	−18.4025	−0.250	0.0461
14%	293	0.170	0.002925	−14.2124	−0.433	0.0470
	313	0.168	0.002891	−15.2132	−0.495	0.0470
	333	0.166	0.002857	−16.2183	−0.560	0.0469
	353	0.171	0.002943	−17.1056	−0.629	0.0466
	373	0.176	0.003028	−17.9856	−0.703	0.0463
	393	0.179	0.003080	−18.8949	−0.780	0.0460
16%	293	0.159	0.002721	−14.3893	−0.488	0.0474
	313	0.157	0.002686	−15.4043	−0.557	0.0474
	333	0.154	0.002635	−16.4419	−0.630	0.0474
	353	0.160	0.002738	−17.3175	−0.708	0.0470
	373	0.166	0.002840	−18.1848	−0.791	0.0467
	393	0.168	0.002874	−19.1209	−0.873	0.0464
18%	293	0.150	0.002552	−14.5448	−0.444	0.0481
	313	0.148	0.002518	−15.5724	−0.507	0.0481
	333	0.147	0.002501	−16.5862	−0.574	0.0480
	353	0.151	0.002569	−17.5038	−0.645	0.0477
	373	0.154	0.002620	−18.4346	−0.720	0.0474
	393	0.159	0.002705	−19.3190	−0.800	0.0471
20%	293	0.140	0.002369	−14.7265	−0.364	0.0490
	313	0.139	0.002352	−15.7503	−0.415	0.0489
	333	0.136	0.002301	−16.8170	−0.470	0.0490
	353	0.140	0.002369	−17.7421	−0.528	0.0487
	373	0.143	0.002420	−18.6818	−0.590	0.0485
	393	0.147	0.002487	−19.5935	−0.655	0.0481

(a) Standard Gibbs free energy (Δ*G^o^_mic_*), (b) standard enthalpy changes of micellization (Δ*H^o^_mic_*), and (c) standard entropy change Δ*S^o^_mic_*, for rhamnolipids in the temperature range of 293–393 K.

These results indicated that changes in the hydrophobicity of the medium play an important role in the micellization process. It was well-established that an increase in temperature which acts as a water breaker decreases the hydrophobic effect, resulting in an increase in CMC of anionic rhamnolipids. The elevation in temperature facilitates interactions between the hydrophobic tails of rhamnolipid molecules. A comparison between the behavior of rhamnolipid in the presence and absence of electrolytes revealed that the extent of decrease in CMC with increased electrolyte concentration was more prominent than those of temperature fluctuations. This observation aligns with the dependency of CMC on the length of the hydrophobic tail of the surfactant. The data showed that the electrolyte addition decreases CMC values by reducing the thickness of the salvation layer around the ionic head of the biosurfactant. Electrostatic repulsive interactions among positive ions of the surfactant decreased, diminishing the hydrophilic property of the surfactant. This enhances its surface and aggregation behavior leading to easy aggregation on the surface and in the solution ultimately resulting in a decrease in CMC values.

### 3.6 Thermodynamic study

The aggregation behavior of rhamnolipids was investigated in order to find out the interactions between electrolytes and RLs in an aqueous system. Spectrophotometric measurement demonstrated a concentration-dependent relationship with rhamnolipids in aqueous electrolyte solutions across a wide range of temperatures. Micellization induced by rhamnolipids in pure aqueous solutions with NaCl was found to be lower compared to standard values without electrolytes. Electrolyte addition resulted in lowering the repulsion between rhamnose moieties and the counter-ion effect of NaCl likely provided the surface for micellization. The extra hydrophobicity offered by the electrolyte appeared to reduce CMC values. However, an increase in CMC values with rising temperature is related to the increase in thermal motion and solubility of hydrophilic groups aiding in micellization. CMC values of rhamnolipids were calculated at temperatures and electrolyte concentrations providing the basis for evaluating thermodynamic parameters to attain further insights into the interactions of rhamnolipids. RL molecules showed excellent structural and micellar stability under different temperatures and varying NaCl concentrations making them ideal candidates for various industrial applications such as enhanced oil recovery, bioremediation of hydrocarbon contaminated sites, and as superior antimicrobial agents. These properties were based on the phase behavior of the RL molecules and how they interact at air–water interfaces. These tendencies were determined by understanding the thermodynamic parameters of the rhamnolipid adsorption and micellization process. RL molecules undergo various structural transformations because of changing concentration, temperature, and electrolytes. It was confirmed from the data that an increase in temperature first decreases the CMC values but a further increase in temperature causes an increase in CMC. The polar hydrophilic heads of rhamnolipids solvated by solvent molecules in their monomeric form disfavor the micellization. Due to the rise in temperature, the de-solvation of hydrophilic heads of rhamnolipid molecules occurs which liberates the monomers and enhances the hydrophobic interactions between the monomers and favors the micellization thus the CMC decreases. However, a further increase in temperature causes an increased kinetic energy of solvent molecules organized around the hydrophobic units which leads to the disruption of this coaxial solvent layer around the hydrophobic part and reduces the micellization. Thus, an increase in CMC values is observed at high temperatures.

The standard Gibbs free energy of micellization, Δ*G^o^_mic_*, was calculated using Equation 1:


(1) $$\Delta {G^{o}}_{\mathrm{mic}}=RT\;\mathrm{In}(X_{CMC)}$$

where X_CMC_ is the mole fraction at which the CMC occurs. R is the gas constant, and T is the temperature in Kelvin.

The standard enthalpy change, Δ*H^o^_mic_*, for micellization, was obtained through the Van’t Hoff relation (Equation 2): (Lang and Wagner, 2017; El-Housseiny et al., 2020; Al Amin Hossain et al., 2022).


(2) $$\Delta {H^{o}}_{\mathrm{mic}}=-{RT}^{2}\;[dIn(X_{CMC}/dT]$$

where [dIn (X_CMC_)/dT] is the slope of the straight line obtained by plotting In(X_CMC_) against temperature.

The standard entropy of micellization, Δ*S^o^_mic_*, was calculated from the relationship in Equation 3.


(3) $$\Delta {G^{o}}_{\mathrm{mic}}=\Delta {H^{o}}_{–c}-T\;\Delta {S^{o}}_{\mathrm{mic}}$$

The negative values of Δ*G^o^_mic_* and Δ*H^o^_mic_* along with positive values of Δ*S^o^_mic_* indicate electrolyte–rhamnolipid interactions. The negative values suggest that the micellization process is generally spontaneous over the entire temperature range and is not energy driven. The decrease in enthalpy Δ*H^o^_mic_* and entropy Δ*S^o^_mic_* with increasing temperature suggested that micellization tends to be an energy-releasing process at high temperatures. This automatically compensates for the contribution of Δ*H^o^_mic_* and Δ*S^o^_mic_* resulting in a consistently negative standard Gibbs free energy (Δ*G^o^_mic_* < 0) dependent on temperature. The values of thermodynamic parameters of micellization for rhamnolipids are listed in Table 2. As the temperature of the system increases from 293 K to 393 K, the Gibbs free energy and enthalpy become more negative, indicating that the process of rhamnolipid micellization was exothermic and becomes more spontaneous and thermodynamically favorable at high temperatures above 373 K. This indicates strong interactions between rhamnolipids and electrolytes. The positive entropy values across all temperatures shown in Table 2 further confirm the spontaneous nature of micellization. However, a slight decrease in entropy was observed at elevated temperatures (373 K and 393 K) attributed to the higher thermal energy available to the system which disrupts hydrogen bonds between solvent molecules, de-solvates the monomeric rhamnose heads of rhamnolipids, and allows the hydrophobic tails to organize into more ordered compact and stable micellar forms. After micelle formation, both the rhamnolipids and solvent molecules rearrange into a more ordered configuration. Thus, a decrease in randomness leads to a decrease in entropy. It is concluded that increasing temperature enhances the spontaneity of rhamnolipid micellization by providing sufficient energy to form new molecular interactions and overcome unfavorable forces.

It was demonstrated that interaction processes within the RL system were temperature dependent, as temperature increases from 293 K to 393 K, hydrophobic hydration weakens, reducing water’s structural order around the hydrophobic tails of rhamnolipids molecules. The increase in temperature causes a distortion in water molecules, which leads to a reduction in the hydration shell around surfactant head groups. At higher temperatures, 373 K and 393 K, the increased molecular motion leads to less organized water structures favoring micellization through hydrophobic interactions. The results indicate the strong London dispersion interactions, a major attractive force for micellization between rhamnolipids and electrolyte NaCl with exothermicity becoming more pronounced at higher temperatures above 373 K. As the concentration of electrolyte varies from 2 to 20% NaCl, both enthalpy and entropy of micellization decreased with the rise in temperature. This behavior can be evidenced at low temperatures 293 K as the reduction of hydrophobic hydration is responsible for the observed increase in standard entropy change. However, as the temperature increased, the structure of water molecules distorted, aggregate molecule sizes decreased, and Gibbs free energy became more exothermic. It was noted that the entropy was dominating the micellization process with Gibbs free energy becoming more negative with the increase in electrolyte concentration up to 20% and temperature to 373 K. The micellization of rhamnolipids was found to be more favorable in the presence of electrolytes. The U-shaped trend noted in the temperature range implies that CMC decreases with the increase in electrolyte concentration. With the increase in temperature, the hydrophobic effect also increased resulting in the penetration of electrolytes in the rhamnolipid micelles.

### 3.7 Antibacterial activity of rhamnolipids

**Figure 7:**
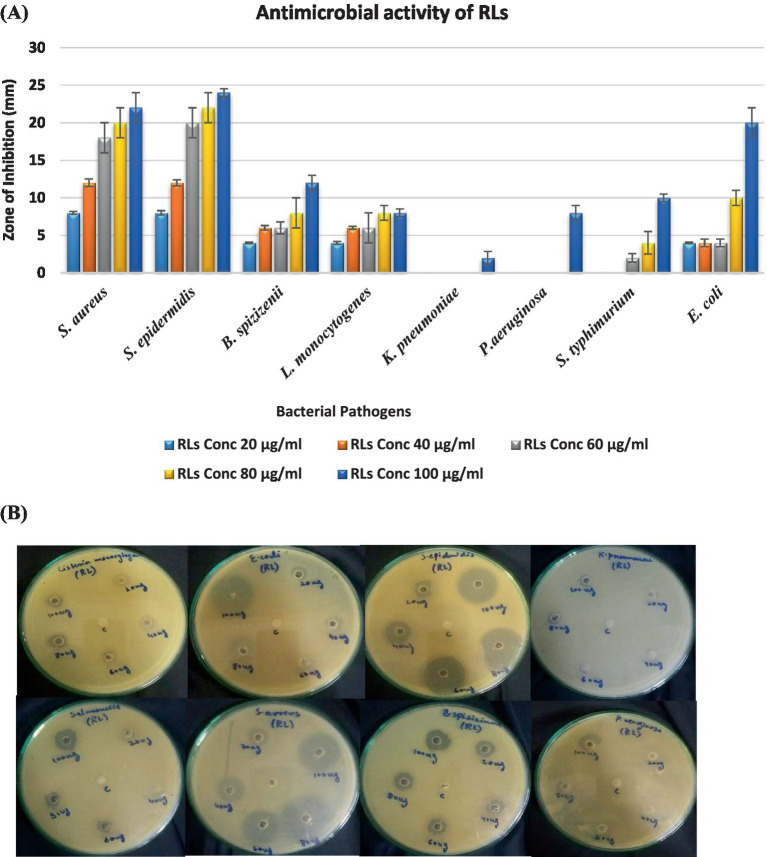
**(A)** Antibacterial activity of RLs against ATCC strains **(B)** Antibacterial activity of rhamnolipids (20, 40, 60, 80, and 100 μg/mL) prepared in methanol solvent (control) from *P. aeruginosa* against ATCC strains.

The antibacterial efficacy of rhamnolipids derived from *P. aeruginosa* was investigated against various ATCC bacterial strains (Figure 7). The findings revealed substantial antibacterial activity, indicating effectiveness against both Gram-positive and negative strains with Gram-positive more significant than Gram-negative. The antimicrobial activity exhibited a direct correlation with the concentration of rhamnolipids which agreed with other studies. As shown in Figure 7, an increase in concentration led to an increment in the zone of inhibition. The result exhibited that *S. aureus* showed a significant zone of inhibition with all the RL concentrations. It showed a maximum zone of inhibition of 22 mm with an RL concentration of 100 μg/mL. *S. epidermidis* showed a zone of inhibition of 20 mm, 22 mm, and 24 mm with RL concentrations of 60 μg/mL, 80 μg/mL, and 100 μg/mL, respectively. *B. spizizenii* exhibited a maximum inhibitory zone of 12 mm with 100 μg/mL of RLs. *K. pneumoniae* and *P. aeruginosa* did not show any zone of inhibition, suggesting they are resistant to RLs. *S. typhimurium* showed a minimal inhibitory zone of 10 mm with a high concentration of 100 μg/mL of RLs. *E. coli* also showed a significant zone of inhibition of 20 mm with 100 μg/mL of RLs.

### 3.8 Antibacterial activity of rhamnolipids in the presence of NaCl

**Figure 8:**
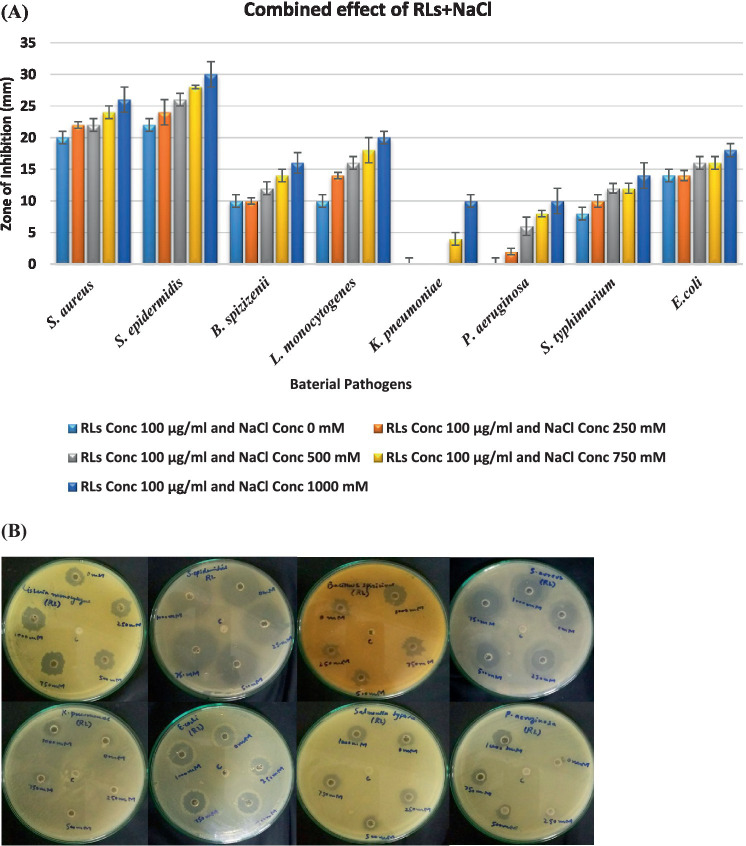
**(A)** Synergistic effect of rhamnolipids and electrolyte on antibacterial activity; **(B)** Antibacterial activity of extracted crude rhamnolipids (100 µg) from *P. aeruginosa* against ATCC strains in the presence of NaCl (0 mM, 250 mM, 500 mM, 750 mM, and 1000 mM). c=control is 1000 mM NaCl.

The combined effect of rhamnolipids and NaCl was analyzed for the same bacteria as shown in Figure 8. Gram-positive strains demonstrated enhanced combined antibacterial activity in the presence of electrolytes. In combination with electrolytes, the antimicrobial potential of the surfactant was doubled. Rhamnolipids exhibit antimicrobial activities mainly because of their distinctive physicochemical characteristics, e.g., their inherent hydrophobic nature, facilitating easy accessibility to cells. The combined effect of 100 μg/mL RLs with different NaCl concentrations enhanced the antibacterial activity. *S. aureus* exhibited zones of inhibition ranging from 22 mm at 250 mM NaCl to 26 mm at 1000 mM NaCl. *S. epidermidis* showed inhibitory zones ranging from 24 mm at 250 mM NaCl to 30 mm at 1000 mM NaCl. *B. spizizenii* showed zones of inhibition from 10 mm to 20 mm at 250 mM to 1,000 mM of NaCl, respectively. Gram-negative bacteria showed quite good zones of inhibition when RLs were combined with varying NaCl concentrations compromising membrane integrity. *K. pneumoniae* showed an inhibitory zone of 10 mm at 1000 mM NaCl. *P. aeruginosa* also exhibited zones of inhibition from 4 mm to 10 mm with 250–1,000 mM NaCl. *S. typhimurium* showed the highest inhibition zones ranging from 10 to 14 mm with 250–1,000 mM NaCl.

## 4 Discussion

Biosurfactants represent an innovative and environmentally friendly alternative to their chemical counterparts, offering several advantages such as biodegradability, stability, environmental compatibility, low toxicity, and a broad range of biological activities (Sanjivkumar et al., 2021; Jahan et al., 2020; El-Housseiny et al., 2020). These characteristics make biosurfactants one of the most versatile processed chemicals with significant potential for future applications, aligning with global sustainability goals of green synthesis and production. Therefore, the search for natural or biosurfactants has increased exponentially in recent years (Liley et al., 2017; Rodrigues et al., 2017; Kopalle et al., 2022). Biological amphiphiles are among those natural compounds that display very unique patterns of self-association generating a fresh wave of knowledge that is likely to create enough opportunity to expand their role in some novel applications (Xu et al., 2018; Chen et al., 2017). The thermodynamic and physicochemical stability of RL micelles, particularly their ability to maintain structural integrity under high temperatures (up to 120°C) and varying NaCl concentrations (up to 20%), makes them ideal for industrial processes like enhanced oil recovery, bioremediation, and pharmaceutical applications (Zdziennicka and Jańczuk, 2017; Zdziennicka et al., 2023). Their potential for biofilm formation, surface tension reduction, and antimicrobial properties, especially in high-temperature environments further underline their utility (Chen, 2004; Azevedo et al., 2021). Significant advancements have also been made in understanding the thermodynamics of RL micellization, contributing to their broader application in diverse fields.

The effect of temperature on the micellization of anionic rhamnolipids is complex. For instance, in the case of synthetic anionic surfactants like SDS, the CMC values initially decrease with increasing temperature but eventually begin to rise (Taj Muhammad and Khan, 2017; Mańko et al., 2014). This irregularity arises from the competition between two opposing effects. On the one hand, as temperature increases, the hydration of the surfactant’s hydrophilic group decreases leading to the so-called “cloud point phenomenon” where the de-solvation (or dehydration) of the hydrophilic portion of the surfactant occurs. This de-solvation effect makes it easier for the surfactant molecules to leave the aqueous phase favoring micelles formation and resulting in a lower CMC. On the other hand, increasing the temperature also causes increased disorder in the structure of the hydrophilic portion that interacts with water. As the structure becomes more disordered, the attachment between polar and non-polar regions weakens reducing the driving force that pushes the hydrophobic tails out of the aqueous phase. This competition between de-solvation and disordering effects leads to the non-linear relationship between temperature and CMC (Zdziennicka and Jańczuk, 2017; Renfro et al., 2014). The recorded values are in close agreement with those found in the literature illustrating a U-shaped trend. The effect of temperature on the micellization of anionic rhamnolipids is quite complex. Understanding these temperature-induced changes in biosurfactant behavior is crucial for optimizing their applications in various contexts. The interaction between surfactants and electrolytes is important due to their widespread domestic and industrial applications. The surface properties of biosurfactants underwent changes with the interaction of electrolytes in the fluid dynamic system. The self-association phenomenon of biosurfactant molecules into organized structures such as micelles, vesicles, and microemulsions is a fundamental property of surface-active metabolites (Anvari et al., 2015; Aranda et al., 2007).

Additionally, the CMC of RLs can be further lowered by the addition of salt to the surfactant solution. In general, the head groups of anionic rhamnolipids show the force of repulsion and resistance against the combination. However, with the addition of NaCl, these repulsive forces are reduced due to the electrostatic shielding effect, which results in the formation of micelles at lower CMC. Further increase in electrolyte concentration reduces electrostatic attraction between ions and micelles, which results in compression of the electrical double layer. Cl^−^ ions bind closely with rhamnolipid ions and decrease the surface charge which further reduces the CMC. The combined effect of increased temperature and salt concentration with rhamnolipid in lowering CMC has opened new avenues in environmental and industrial applications. The unique physiochemical properties and micellization behavior of rhamnolipids make them suitable for use in bioremediation, enhancing bioavailability, bio-degradation processes, and reducing heavy metals in the environment (Jahan et al., 2020; Guzmán et al., 2024).

The negative values of the standard Gibbs free energy of micellization show that the solubilization and interaction of rhamnolipid is an exothermic process reflecting strong interactions between rhamnolipids and electrolytes. As the electrolyte concentration increases, the standard enthalpy and entropy of micellization decrease with the rise in temperature (Mishra et al., 2023). This behavior suggests that at lower temperatures the reduction in hydrophobic hydration is responsible for the observed increase in the standard entropy change (Δ*S^o^_mic_*). However, with the increase in temperature, the structure of water molecules becomes more distorted, and the size of aggregate molecules also decreases resulting in a more exothermic Gibbs free energy with this effect becoming more pronounced. The negative Δ*H^o^_mic_* values provide evidence that London dispersion interaction represents the primary attractive force for micellization with an increase in temperature. As a result, the contribution to Gibbs free energy also increases implying that hydrogen bonds between water molecules weaken requiring less energy for the disruption of water molecule clusters. The magnitude of Δ*H^o^_mic_* becomes more significant at higher temperatures, further supporting the exothermic nature of the process. In all cases, the positive entropy change confirms that the micellization behavior of rhamnolipids is favored entropically (Zhang et al., 2022). As monomeric surfactant molecules aggregate to form micelles the positive entropy change indicates the randomness of the hydrocarbon tails of rhamnolipid monomers within the micellar core. However, as temperature increases Δ*S^o^_mic_* decreases resulting in reduced self-aggregation because of greater molecular motion at elevated temperatures. The Gibbs free energy change is the sum of standard enthalpy and standard entropy changes. The negative standard Gibbs free energy demonstrates that micellization is spontaneous and this spontaneity increases with higher electrolyte concentrations and temperatures. It is noted that entropy dominates over the micellization process within the system (Mishra et al., 2023). The calculated thermodynamic parameters confirm that in all cases Δ*G^o^_mic_* remains negative and becomes more negative with the increase in electrolyte concentration and temperature. This indicates that the micellization of rhamnolipid is more favorable in the presence of an electrolyte. A U-shaped trend was noted across the temperature range where the CMC decreases with the increase of electrolyte concentration. As the temperature increases, the hydrophobic effect also increases facilitating the penetration of electrolytes into the rhamnolipid micelles. Increasing the salt concentration reduces the CMC of rhamnolipids while making Gibbs free energy and enthalpy more negative and increasing entropy. These thermodynamic changes show that rhamnolipid micellization becomes more spontaneous with higher salt concentrations. Rhamnolipids an anionic biosurfactant have negatively charged head groups. When these surfactants arrange themselves into a micellar form the repulsion between their negatively charged head groups counteracts the micellization process and enhances its CMC. The addition of NaCl to the surfactant solution provides counter-ions that neutralize the negative charge on the rhamnolipids head groups thereby reducing the repulsive forces (Kumar et al., 2012). As a result, micellization becomes more favorable. This reduction in repulsion explains the observed decrease in CMC values as well as the more negative Gibbs free energy and enthalpy values with increasing salt concentration (Umlong and Ismail, 2007). The contribution to the Gibbs free energy also increased, suggesting that hydrogen bonds between water molecules started weakening thereby, less energy was required for the breakup of the water molecule clusters. The Δ*H^o^_mic_* became more significant at higher temperatures. The entropy change was positive in all cases which proved that the aggregation behavior of rhamnolipids was favored entropically. The monomeric surfactant molecules started to form aggregate structures, i.e., micelles. The positive value of entropy change indicated the cluster around the hydrocarbon tails of the rhamnolipid monomer and increased the randomness of the hydrocarbon chain in the micellar core. The values of Δ*S^o^_mic_* decreased with the rise of temperature resulting in poor self-aggregation because of the increased molecular motion at higher temperatures. The entropy of micellization was positive throughout the temperature range representing that the micellization process was endothermic because the head group was more hydrated than the hydrophobic tail with increased temperature. This led to an overall ordering of the system lowering entropy with increases in temperature. Δ*G^o^_mic_*, the sum of standard enthalpy and standard entropy change was consistently negative indicating a significant spontaneous micellization (Umlong and Ismail, 2007).

The antimicrobial activity of rhamnolipids is because of the physicochemical characteristics of rhamnolipid homologs. These biosurfactants are highly effective in antimicrobial applications due to their hydrophobic nature which makes them easily interact with cell membranes (Rahman et al., 2010; Sotirova et al., 2012). The remarkable physicochemical characteristics of rhamnolipids make these compounds attractive alternatives to traditional antibiotics. The chemical composition of the rhamnolipid mixture has significant impacts on antimicrobial activities (El-Sheshtawy and Doheim, 2014). The results illustrated that Gram-positive bacteria are more prone to antibacterial activities governed by RLs which was also confirmed from different studies (de Freitas Ferreira et al., 2019). The Gram-negative strains are generally resistant to RL because of outer membrane protection and stability (Zhang et al., 2017). Minor zone of inhibition showed by *S. typhae* and *E. coli* with 100 μg/mL of RL showed the possibility of concentration-dependent inhibition. It could be possible that when the RL concentration was increased the Gram-negative bacteria also exhibited a significant zone of inhibition. The results suggested that when different NaCl concentrations were added along with RL there was increased antibacterial activity. Gram-negative bacteria also showed a significant zone of inhibition, highlighting the potential of RLs and NaCl combinations as effective antimicrobial agents (Rodrigues et al., 2017). It is generally accepted that biosurfactants primarily affect cell membrane integrity (Deepika et al., 2015). However, the mechanism of action varies depending on the specific microorganism (Zhao et al., 2022). Rhamnolipids increase the cell membrane permeability by disrupting it or forming bonds with membrane phospholipids. They can also increase the conductance of the membrane by accumulating intercellular particles acting on both the protein and lipid components to induce structural changes (Bharali et al., 2013; Rodrigues et al., 2017). These alterations in cell membrane physiology lead to cell lysis. As a result, rhamnolipids function similarly to antibiotics by bonding with the surface membrane components of bacteria and compromising their integrity (Zhao et al., 2013; Dusane et al., 2010). Because of these characteristic features, rhamnolipids hold promise in diverse biomedical fields. Rhamnolipids of microbial origin present a significant alternative to toxic synthetic surfactants with potential applications in environmental fields and biotechnology (Tiso et al., 2017; El-Housseiny et al., 2020). However, their efficient utilization is blundered by a limited understanding of their micellization process in aqueous media. Rhamnolipids exhibit high tolerance to extreme salt concentrations like the synthetic anionic surfactant SDS. It was found that RLs produced by *P. aeruginosa* consist of a mixture of homologs containing a number of congeners with di-rhamnolipids being predominant in our study. This variation of congener composition causes fluctuation in surface activity and micellization behavior in fluid dynamic systems. Understanding the surface-active and self-assembly properties of rhamnolipids makes them promising candidates for utilization in pharmaceuticals, consumer products, and environmental applications.

## 5 Conclusion

Rhamnolipids, a novel subclass of glycolipids, show remarkable stability under extreme conditions due to their unique structural and physiochemical properties. Their variation in structures, aggregation behaviors, interaction patterns, and phase transition, influenced by thermal effects and NaCl concentrations, open new avenues in nanomedicines and enhance their antimicrobial potential. Thermodynamic studies show entropy-driven micellization, providing insights into molecular integrations and structural dynamics. This study has explored the micellization properties and behavior of rhamnolipids in response to varying temperatures (293–393 K) and (2–20%) electrolyte concentrations. The results underline the importance of these factors in determining the aggregation behavior with CMC values following a U-shape trend. The demonstrated stability of rhamnolipids under harsh conditions (elevated temperatures, extreme pH, and high salt concentrations) highlights their potential for applications in industrial operations in challenging environments. Additionally, the surfactants also exhibit enhanced antimicrobial effects against Gram-positive pathogens (*S. aureus*, *S. epidermidis,* and *L. monocytogene*), with zones of inhibition of 26, 30, and 20 mm in the presence of NaCl. These findings enhance the understanding of the molecular integration and structural evolution of rhamnolipids and position them as valuable candidates for replacing synthetic surfactants in industries requiring robust performance under extreme conditions, thereby significantly contributing to the advancement of biosurfactant research.

## Data Availability

The raw data supporting the conclusions of this article will be made available by the authors, without undue reservation.
